# Effectiveness of Monovalent mRNA Vaccines Against COVID-19–Associated Hospitalization Among Immunocompetent Adults During BA.1/BA.2 and BA.4/BA.5 Predominant Periods of SARS-CoV-2 Omicron Variant in the United States — IVY Network, 18 States, December 26, 2021–August 31, 2022

**DOI:** 10.15585/mmwr.mm7142a3

**Published:** 2022-10-21

**Authors:** Diya Surie, Levi Bonnell, Katherine Adams, Manjusha Gaglani, Adit A. Ginde, David J. Douin, H. Keipp Talbot, Jonathan D. Casey, Nicholas M. Mohr, Anne Zepeski, Tresa McNeal, Shekhar Ghamande, Kevin W. Gibbs, D. Clark Files, David N. Hager, Arber Shehu, Anne P. Frosch, Heidi L. Erickson, Michelle N. Gong, Amira Mohamed, Nicholas J. Johnson, Vasisht Srinivasan, Jay S. Steingrub, Ithan D. Peltan, Samuel M. Brown, Emily T. Martin, Akram Khan, William S. Bender, Abhijit Duggal, Jennifer G. Wilson, Nida Qadir, Steven Y. Chang, Christopher Mallow, Carolina Rivas, Jennie H. Kwon, Matthew C. Exline, Adam S. Lauring, Nathan I. Shapiro, Natasha Halasa, James D. Chappell, Carlos G. Grijalva, Todd W. Rice, William B. Stubblefield, Adrienne Baughman, Kelsey N. Womack, Kimberly W. Hart, Sydney A. Swan, Yuwei Zhu, Jennifer DeCuir, Mark W. Tenforde, Manish M. Patel, Meredith L. McMorrow, Wesley H. Self, IVY Network, IVY Network, Nicole Calhoun, Judy Herrick, Eric Hoffman, Amanda McKillop, Kempapura Murthy, Michael Smith, Martha Zayed, Lesley De Souza, Lori-Ann Kozikowski, Scott Ouellette, Kiran Ashok, Susan Gole, Alexander King, Omar Mehkri, Bryan Poynter, Caitlin ten Lohuis, Nicholas Stanley, Sean Caspers, Audrey Hendrickson, Olivia Kaus, Leyla Taghizadeh, Walker Tordsen, Valerie Aston, Robert Bowers, Jeffrey Jorgensen, Jennifer King, Harith Ali, Richard E. Rothman, Jen-Ting Chen, Rahul Nair, Gopal Allada, Genesis Briceno, Shewit Giovanni, Kinsley A. Hubel, Jesus Martinez, Minn Oh, Jonathan Pak, Jose Pena, Alexandra Jun Gordon, Joe Levitt, Cynthia Perez, Jonasel Roque, Anita Visweswaran, Sarah Karow, Maryiam Khan, Austin Klingler, Sarah Pannu, David Smith, Elizabeth Schwartz, Connor Snyder, Madison So, Preston So, Gabrielle Swoope, Michael Weigand, Michael Carricato, Ian Chambers, Conner Driver, Jennifer Goff, David Huynh, Kelly Jensen, Sukantha Chandrasekaran, Trevor Frankel, Omai Garner, Catherine Fairfield, Shannon Landers, Paul Nassar, Cameron Williams, Hayley Gershengorn, Ramsay Bielak, Christopher Blair, William J. Fitzsimmons, Rebecca Fong, Julie Gilbert, EJ McSpadden, Lara Thomas, Rachel Truscon, Weronika Damek Valvano, Layla A. Anderson, Christine D. Crider, Thomas C. Paulson, Kyle A. Steinbock, Marica Blair, Lauren J. Ezzell, Samarian J. Hargrave, Christy Kampe, Jakea Johnson, Jennifer L. Luther, Rendie E. McHenry, Bryan P. M. Peterson, Claudia Guevara Pulido, Laura L. Short, Margaret E. Whitsett, Madeline Hicks, Leigha Landreth, Mary LaRose, Lisa Parks, Hilary Babcock, Tiffany Hink, Kevin Jolani, David McDonald, Caroline O’Neal, Bijal Parikh, Katie Parrish, Carleigh Samuels

**Affiliations:** ^1^CDC COVID-19 Emergency Response Team; ^2^Baylor Scott & White Health, Temple, Texas; ^3^Texas A&M University College of Medicine, Temple, Texas; ^4^University of Colorado School of Medicine, Aurora, Colorado; ^5^Vanderbilt University Medical Center, Nashville, Tennessee; ^6^University of Iowa, Iowa City, Iowa; ^7^Wake Forest University Baptist Medical Center, Winston-Salem, North Carolina; ^8^Johns Hopkins Hospital, Baltimore, Maryland; ^9^Hennepin County Medical Center, Minneapolis, Minnesota; ^10^Montefiore Healthcare Center, Albert Einstein College of Medicine, New York, New York; ^11^University of Washington School of Medicine, Seattle, Washington; ^12^Baystate Medical Center, Springfield, Massachusetts; ^13^Intermountain Medical Center and University of Utah, Salt Lake City, Utah; ^14^University of Michigan School of Public Health, Ann Arbor, Michigan; ^15^Oregon Health & Science University Hospital, Portland, Oregon; ^16^Emory University School of Medicine, Atlanta, Georgia; ^17^Cleveland Clinic, Cleveland, Ohio; ^18^Stanford University School of Medicine, Stanford, California; ^19^Ronald Reagan UCLA Medical Center, Los Angeles, California; ^20^University of Miami, Miami, Florida; ^21^Washington University, St. Louis, Missouri; ^22^The Ohio State University Wexner Medical Center, Columbus, Ohio; ^23^University of Michigan School of Medicine, Ann Arbor, Michigan; ^24^Beth Israel Deaconess Medical Center, Boston, Massachusetts.; Baylor Scott & White Health; Baylor Scott & White Health; Baylor Scott & White Health; Baylor Scott & White Health; Baylor Scott & White Health; Baylor Scott & White Health; Baylor Scott & White Health; Baystate Medical Center; Baystate Medical Center; Baystate Medical Center; Cleveland Clinic; Cleveland Clinic; Cleveland Clinic; Cleveland Clinic; Cleveland Clinic; Emory University; Emory University; Hennepin County Medical Center; Hennepin County Medical Center; Hennepin County Medical Center; Hennepin County Medical Center; Hennepin County Medical Center; Intermountain Medical Center; Intermountain Medical Center; Intermountain Medical Center; Intermountain Medical Center; Johns Hopkins University; Johns Hopkins University; Montefiore Medical Center; Montefiore Medical Center; Oregon Health & Science University; Oregon Health & Science University; Oregon & Health Science University; Oregon Health & Science University; Oregon Health & Science University; Oregon Health & Science University; Oregon Health & Science University; Oregon Health & Science University; Stanford University; Stanford University; Stanford University; Stanford University; Stanford University; The Ohio State University; The Ohio State University; The Ohio State University; The Ohio State University; The Ohio State University; The Ohio State University; The Ohio State University; The Ohio State University; The Ohio State University; The Ohio State University; The Ohio State University; UCHealth University of Colorado Hospital; UCHealth University of Colorado Hospital; UCHealth University of Colorado Hospital; UCHealth University of Colorado Hospital; UCHealth University of Colorado Hospital; UCHealth University of Colorado Hospital; University of California, Los Angeles; University of California, Los Angeles; University of California, Los Angeles; University of Iowa; University of Iowa; University of Iowa; University of Iowa; University of Miami; University of Michigan; University of Michigan; University of Michigan; University of Michigan; University of Michigan; University of Michigan; University of Michigan; University of Michigan; University of Michigan; University of Washington; University of Washington; University of Washington; University of Washington; Vanderbilt University Medical Center; Vanderbilt University Medical Center; Vanderbilt University Medical Center; Vanderbilt University Medical Center; Vanderbilt University Medical Center; Vanderbilt University Medical Center; Vanderbilt University Medical Center; Vanderbilt University Medical Center; Vanderbilt University Medical Center; Vanderbilt University Medical Center; Vanderbilt University Medical Center; Wake Forest University; Wake Forest University; Wake Forest University; Wake Forest University; Washington University; Washington University; Washington University; Washington University; Washington University; Washington University; Washington University; Washington University.

The SARS-CoV-2 Omicron variant (B.1.1.529 or BA.1) became predominant in the United States by late December 2021 (*1*). BA.1 has since been replaced by emerging lineages BA.2 (including BA.2.12.1) in March 2022, followed by BA.4 and BA.5, which have accounted for a majority of SARS-CoV-2 infections since late June 2022 (*1*). Data on the effectiveness of monovalent mRNA COVID-19 vaccines against BA.4/BA.5-associated hospitalizations are limited, and their interpretation is complicated by waning of vaccine-induced immunity (*2*–*5*). Further, infections with earlier Omicron lineages, including BA.1 and BA.2, reduce vaccine effectiveness (VE) estimates because certain persons in the referent unvaccinated group have protection from infection-induced immunity. The IVY Network^†^ assessed effectiveness of 2, 3, and 4 doses of monovalent mRNA vaccines compared with no vaccination against COVID-19–associated hospitalization among immunocompetent adults aged ≥18 years during December 26, 2021–August 31, 2022. During the BA.1/BA.2 period, VE 14–150 days after a second dose was 63% and decreased to 34% after 150 days. Similarly, VE 7–120 days after a third dose was 79% and decreased to 41% after 120 days. VE 7–120 days after a fourth dose was 61%. During the BA.4/BA.5 period, similar trends were observed, although CIs for VE estimates between categories of time since the last dose overlapped. VE 14–150 days and >150 days after a second dose was 83% and 37%, respectively. VE 7–120 days and >120 days after a third dose was 60%and 29%, respectively. VE 7–120 days after the fourth dose was 61%. Protection against COVID-19–associated hospitalization waned even after a third dose. The newly authorized bivalent COVID-19 vaccines include mRNA from the ancestral SARS-CoV-2 strain and from shared mRNA components between BA.4 and BA.5 lineages and are expected to be more immunogenic against BA.4/BA.5 than monovalent mRNA COVID-19 vaccines (*6*–*8*). All eligible adults aged ≥18 years^§^ should receive a booster dose, which currently consists of a bivalent mRNA vaccine, to maximize protection against BA.4/BA.5 and prevent COVID-19–associated hospitalization.

During December 26, 2021–August 31, 2022, adults aged ≥18 years admitted for COVID-19–like illness^¶^ within the IVY Network of 21 hospitals in 18 states were eligible for inclusion in this test-negative, case-control analysis. Among patients hospitalized with COVID-19–like illness, case-patients received a positive SARS-CoV-2 nucleic acid amplification test (NAAT) or antigen test result within 14 days of illness onset and control-patients received a negative SARS-CoV-2 NAAT result. Upper respiratory specimens were collected from all enrolled patients and tested by reverse transcription–polymerase chain reaction (RT-PCR) at a central laboratory (Vanderbilt University Medical Center, Nashville, Tennessee). Specimens testing positive for SARS-CoV-2 were sent to the University of Michigan (Ann Arbor, Michigan) for whole genome sequencing to determine SARS-CoV-2 lineages.** Periods of lineage predominance were defined based on when >50% of sequenced specimens within the IVY Network represented a particular lineage.

Demographic and clinical data were obtained through electronic medical record (EMR) review and patient (or proxy) interview. COVID-19 mRNA vaccination status was verified from EMRs, state-based registries, vaccination cards, or self-report and adjudicated based on vaccination dates. Four vaccination groups were defined: 1) patients who received no vaccine doses before illness onset, 2) patients who received 2 doses of a monovalent mRNA vaccine ≥14 days before illness onset, 3) patients who received 3 doses of a monovalent mRNA vaccine ≥7 days before illness onset, and 4) patients who received 4 doses of a monovalent mRNA vaccine ≥7 days before illness onset. Patients were excluded if they had an immunocompromising condition,^††^ had an incomplete vaccination series, or had received a non-mRNA vaccine.^§§^

VE to prevent COVID-19–associated hospitalization was estimated by comparing the odds of antecedent monovalent mRNA vaccination (2, 3, or 4 doses) versus no previous vaccination between case-patients and control-patients. Using multivariable logistic regression models, VE was calculated as (1 − adjusted odds ratio [aOR]) × 100. Models were adjusted for U.S. Department of Health and Human Services region, calendar time in biweekly intervals, age group (18–49, 50–64, and ≥65 years), sex, race, and Hispanic or Latino (Hispanic) ethnicity. Results were stratified by periods of Omicron variant predominance (i.e., December 26, 2021–June 19, 2022 [BA.1/BA.2 period] and June 20–August 31, 2022 [BA.4/BA.5 period]), and by days since the last monovalent vaccine dose (14–150 days versus >150 days for 2 doses and 7–120 versus >120 days for 3 or 4 doses to align with previous guidance for next dose eligibility).^¶¶^ Differences with nonoverlapping 95% CIs were considered to be statistically significant. Analyses were conducted using Stata (version 17; StataCorp). This activity was determined to be public health surveillance by each participating site and CDC and was conducted consistent with applicable federal law and CDC policy.***

During December 26, 2021–August 31, 2022, a total of 6,599 immunocompetent patients were enrolled in the IVY Network, and 4,730 (72%) adult patients were included in the analysis (Table 1) (Figure). (A total of 1,869 patients were excluded from this analysis for the following reasons: non-mRNA vaccine receipt [390]; partially vaccinated [158]; implausible or unverified vaccination dates [632]; received vaccination before CDC recommendations [169]; illness onset >10 days before test date [125]; illness onset >14 days before hospitalization [12]; missing data [274]; withdrew [nine]; other [100].) Among the 4,730 patients included, 3,352 (71%) were enrolled during the BA.1/BA.2 period (1,699 case-patients and 1,653 control-patients) and 1,378 (29%) during the BA.4/BA.5 period (707 case-patients and 671 control-patients).

**TABLE 1:** Characteristics of immunocompetent adults hospitalized during BA.1/BA.2 and BA.4/BA.5 predominant periods of SARS-CoV-2 Omicron variant circulation* — IVY Network, 21 hospitals^†^ in 18 U.S. states, December 26, 2021–August 31, 2022

Characteristic	No. (%)				
	Total (N = 4,730)	BA.1/BA.2 period		BA.4/BA.5 period	
		COVID-19 case-patients (n = 1,699)	Test-negative control-patients (n = 1,653)	COVID-19 case-patients (n = 707)	Test-negative control-patients (n = 671)
**Vaccination status, no. of COVID-19 vaccine doses received**					
Unvaccinated	1,513 (32)	709 (42)	435 (26)	214 (30)	155 (23)
2	1,345 (28)	533 (31)	483 (29)	148 (21)	181 (27)
3	1,636 (35)	432 (25)	694 (42)	277 (39)	233 (35)
4	236 (5)	25 (1)	41 (2)	68 (10)	102 (15)
**Female sex**	2,319 (49)	807 (47)	823 (50)	360 (51)	329 (49)
**Median age, yrs (IQR)**	65 (52–76)	65 (52–77)	63 (50–74)	69 (54–79)	64 (54–74)
**Age group, yrs**					
18–49	1,012 (21)	363 (21)	392 (24)	141 (20)	116 (17)
50–64	1,345 (28)	460 (27)	496 (30)	151 (21)	238 (35)
65–74	1,071 (23)	380 (22)	386 (23)	150 (21)	155 (23)
75–84	862 (18)	323 (19)	260 (16)	170 (24)	109 (16)
≥85	440 (9)	173 (10)	119 (7)	95 (13)	53 (8)
**Race or ethnicity**					
Black, non-Hispanic	910 (19)	314 (18)	352 (21)	114 (16)	130 (19)
White, non-Hispanic	2,846 (60)	999 (59)	985 (60)	457 (65)	405 (60)
Hispanic, any race	631 (13)	245 (14)	199 (12)	91 (13)	96 (14)
Other race, non-Hispanic^§^	251 (5)	108 (6)	79 (5)	36 (5)	28 (4)
Other^¶^	92 (2)	33 (2)	38 (2)	9 (1)	12 (2)
**HHS Region**					
1	941 (20)	403 (24)	303 (18)	113 (16)	122 (18)
2	266 (6)	62 (4)	90 (5)	51 (7)	63 (9)
3	153 (3)	59 (3)	62 (4)	17 (2)	15 (2)
4	879 (19)	356 (21)	366 (22)	85 (12)	75 (11)
5	564 (12)	208 (12)	216 (13)	74 (10)	66 (10)
6	486 (10)	116 (7)	136 (8)	121 (17)	113 (17)
7	346 (7)	118 (7)	101 (6)	61 (9)	66 (10)
8	643 (14)	207 (12)	219 (13)	119 (17)	98 (15)
9	174 (4)	67 (4)	63 (4)	24 (3)	20 (3)
10	278 (6)	103 (6)	100 (6)	42 (6)	33 (5)
**No. of underlying conditions**					
0	563 (12)	258 (15)	161 (10)	75 (11)	69 (10)
1	1,223 (26)	445 (26)	413 (25)	200 (28)	165 (25)
2	1,387 (29)	473 (28)	482 (29)	201 (28)	231 (34)
≥3	1,557 (33)	523 (31)	597 (36)	231 (33)	206 (31)

**Abbreviation:** HHS = U.S. Department of Health and Human Services.

* BA.1/BA.2 period was during December 26, 2021–June 19, 2022; BA.4/BA.5 period was during June 20–August 31, 2022.

**^†^** Hospitals by HHS region included *Region 1:* Baystate Medical Center (Springfield, Massachusetts), Beth Israel Deaconess Medical Center (Boston, Massachusetts); *Region 2:* Montefiore Medical Center (New York, New York); *Region 3:* Johns Hopkins Hospital (Baltimore, Maryland); *Region 4:* Emory University Medical Center (Atlanta, Georgia), University of Miami Medical Center (Miami, Florida), Vanderbilt University Medical Center (Nashville, Tennessee), Wake Forest University Baptist Medical Center (Winston-Salem, North Carolina); *Region 5:* Cleveland Clinic (Cleveland, Ohio), Hennepin County Medical Center (Minneapolis, Minnesota), The Ohio State University Wexner Medical Center (Columbus, Ohio), University of Michigan Hospital (Ann Arbor, Michigan); *Region 6:* Baylor Scott & White Health (Temple, Texas); *Region 7:* Barnes-Jewish Hospital (St. Louis, Missouri), University of Iowa Hospitals (Iowa City, Iowa); *Region 8:* Intermountain Medical Center (Murray, Utah), UCHealth University of Colorado Hospital (Aurora, Colorado); *Region 9:* Ronald Reagan UCLA Medical Center (Los Angeles, California), Stanford University Medical Center (Stanford, California); and *Region 10:* Oregon Health & Science University Hospital (Portland, Oregon), University of Washington Medical Center (Seattle, Washington).

^§^ Other race includes Asian, Native American or Alaska Native, and Native Hawaiian or other Pacific Islander, which were combined because of small counts.

^¶^ Self-reported race and ethnicity as other or non-Hispanic, or patients for whom information on race and ethnicity was unavailable.

**FIGURE:**
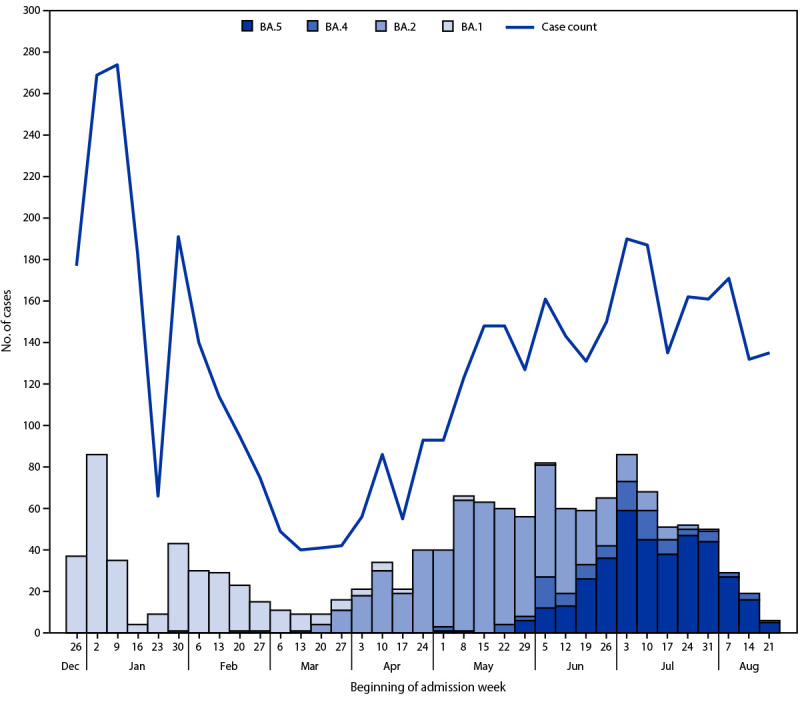
Numbers of COVID-19 cases* and SARS-CoV-2 whole genome–sequenced lineages^†^^,^^§^^,^^¶^ among immunocompetent adults hospitalized with COVID-19 — IVY Network, 21 hospitals in 18 U.S. states,** December 26, 2021–August 24, 2022†† * N = 4,543. ^†^ Number of SARS-CoV-2 whole genome–sequenced lineages: BA.1 = 349; BA.2 = 568; BA.4 = 91; and BA.5 = 376. ^§^ Upper respiratory specimens collected from COVID-19 patients for detection of SARS-CoV-2 by reverse transcription–polymerase chain reaction (RT-PCR) were eligible for whole genome sequencing. During the early BA.1 period (December 26, 2021–January 14, 2022), all specimens testing positive for SARS-CoV-2 by RT-PCR were submitted for whole genome sequencing; from January 15, 2022 onward, only specimens testing positive for SARS-CoV-2 by RT-PCR with a cycle threshold <32 for at least one of two nucleocapsid gene targets tested underwent whole genome sequencing. SARS-CoV-2 lineages were assigned by using PANGO on genomes with >80% coverage. ^¶^ BA.1, BA.2, BA.4, and BA.5 lineages. Among specimens from 568 patients who received test results indicating BA.2 lineage, 343 (60%) indicated BA.2.12.1 lineage. ** Barnes-Jewish Hospital (St. Louis, Missouri), Baylor Scott & White Health (Temple, Texas), Baystate Medical Center (Springfield, Massachusetts), Beth Israel Deaconess Medical Center (Boston, Massachusetts), Cleveland Clinic (Cleveland, Ohio), Emory University Medical Center (Atlanta, Georgia), Hennepin County Medical Center (Minneapolis, Minnesota), Intermountain Medical Center (Murray, Utah), Johns Hopkins Hospital (Baltimore, Maryland), Montefiore Medical Center (New York, New York), Oregon Health & Science University Hospital (Portland, Oregon), Ronald Reagan UCLA Medical Center (Los Angeles, California), Stanford University Medical Center (Stanford, California), The Ohio State University Wexner Medical Center (Columbus, Ohio), UCHealth University of Colorado Hospital (Aurora, Colorado), University of Iowa Hospitals (Iowa City, Iowa), University of Miami Medical Center (Miami, Florida), University of Michigan Hospital (Ann Arbor, Michigan), University of Washington Medical Center (Seattle, Washington), Vanderbilt University Medical Center (Nashville, Tennessee), Wake Forest University Baptist Medical Center (Winston-Salem, North Carolina). ^††^ Sequencing results complete through August 24, 2022. Low numbers of COVID-19 cases and SARS-CoV-2 whole genome–sequenced lineages in late January reflect an administrative pause in IVY Network enrollment during January 25–31, 2022.

Case-patients’ median ages during the BA.1/BA.2 period and the BA.4/BA.5 period were 65 and 69 years, respectively. Among patients enrolled during the BA.1/BA.2 period, 1,144 (34%) were unvaccinated, 1,016 (30%) had received 2 doses, 1,126 (34%) had received 3 doses, and 66 (2%) had received 4 doses. Among 1,378 patients included during the BA.4/BA.5 period, 369 (27%) were unvaccinated, 329 (24%) had received 2 doses, 510 (37%) had received 3 doses, and 170 (12%) had received 4 doses.

During the BA.1/BA.2 period, the overall VE of 3 COVID-19 mRNA vaccine doses against COVID-19–associated hospitalization (median interval between the last dose and illness onset = 145 days) was 69% (Table 2), and during the BA.4/BA.5 period (median interval between the last dose and illness onset = 233 days) was 31%; whereas overall VE of 2 or 4 doses between lineage periods was similar (39% versus 41% for 2 doses and 61% versus 60% for 4 doses). During the BA.1/BA.2 period, VE of 2 doses waned from 63% at 14–150 days since the second dose to 34% at >150 days, VE of 3 doses waned from 79% at 7–120 days since the last dose to 41% at >120 days, and VE of 4 doses 7–120 days after vaccination was 61%. During the BA.4/BA.5 period, VE estimates of 2 doses 14–150 days and >150 days after the second dose were 83% and 37%, respectively; VE estimates of 3 doses 7–120 days and >120 days from the last dose were 60% and 29%, respectively. VE of 4 doses 7–120 days after vaccination was 61%.

**TABLE 2:** Effectiveness of monovalent mRNA vaccines against COVID-19–associated hospitalization during the BA.1/BA.2 and BA.4/BA.5 predominant periods of SARS-CoV-2 Omicron variant circulation* among immunocompetent adults — IVY Network, 21 hospitals in 18 U.S. states,^†^ December 26, 2021–August 31, 2022

Group/No. of doses	Interval from last vaccine dose to illness onset, days^§^	Median interval (IQR) from last vaccine dose to illness, days	Vaccinated case-patients, no./total no. (%)	Vaccinated control-patients, no./total no. (%)	Adjusted VE, % (95% CI)^¶^
**BA.1/BA.2 period**					
2	≥14	277 (216–341)	533/1,242 (43)	483/918 (53)	39 (26–49)
	14–150	111 (87–130)	62/771 (8)	79/514 (15)	63 (46–75)
	>150	290 (241–351)	471/1,180 (40)	404/839 (48)	34 (20–46)
3	≥7	145 (92–190)	432/1,141 (38)	694/1,129 (61)	69 (62–74)
	7–120	80 (55–100)	167/876 (19)	393/828 (47)	79 (74–84)
	>120	180 (154–208)	265/974 (27)	301/736 (41)	41 (23–55)
4	≥7	26 (16–39)	25/734 (3)	41/476 (9)	61 (29–78)
	7–120	26 (16–39)	25/734 (3)	41/476 (9)	61 (29–78)
	>120	—	—	—	—
**BA.4/BA.5 period**					
2	≥14	428 (324–468)	131/317 (41)	181/336 (54)	41 (17–57)
	14–150	102 (77–123)	3/189 (2)	13/168 (8)	83 (35–96)
	>150	430 (329–471)	128/314 (41)	168/323 (52)	37 (12–55)
3	≥7	233 (196–267)	232/418 (56)	232/387 (60)	31 (7–49)
	7–120	74 (33–110)	13/199 (7)	24/179 (13)	60 (12–81)
	>120	237 (204–269)	219/405 (54)	208/363 (57)	29 (3–48)
4	≥7	69 (54–103)	63/249 (25)	102/257 (40)	60 (36–75)
	7–120	66 (51–85)	56/242 (23)	95/250 (38)	61 (37–76)
	>120	131 (126–137)	7/193 (4)	7/162 (4)	—

**Abbreviation**: VE = vaccine effectiveness.

* BA.1/BA.2 period was during December 26, 2021–June 19, 2022; BA.4/BA.5 period was during June 20–August 31, 2022.

**^†^** Hospitals included Barnes-Jewish Hospital (St. Louis, Missouri), Baylor Scott & White Health (Temple, Texas), Baystate Medical Center (Springfield, Massachusetts), Beth Israel Deaconess Medical Center (Boston, Massachusetts), Cleveland Clinic (Cleveland, Ohio), Emory University Medical Center (Atlanta, Georgia), Hennepin County Medical Center (Minneapolis, Minnesota), Intermountain Medical Center (Murray, Utah), Johns Hopkins Hospital (Baltimore, Maryland), Montefiore Medical Center (New York, New York), Oregon Health & Science University Hospital (Portland, Oregon), Ronald Reagan UCLA Medical Center (Los Angeles, California), Stanford University Medical Center (Stanford, California), The Ohio State University Wexner Medical Center (Columbus, Ohio), UCHealth University of Colorado Hospital (Aurora, Colorado), University of Iowa Hospitals (Iowa City, Iowa), University of Miami Medical Center (Miami, Florida), University of Michigan Hospital (Ann Arbor, Michigan), University of Washington Medical Center (Seattle, Washington), Vanderbilt University Medical Center (Nashville, Tennessee), Wake Forest University Baptist Medical Center (Winston-Salem, North Carolina).

**^§^** An interval of 14 days was used to estimate the time needed to acquire immunity after receipt of a primary COVID-19 vaccination series; after the initial priming of the immune system, a shorter interval of 7 days was used to estimate the time required for response to booster doses. A threshold of 150 days was used to assess waning of 2-dose VE because eligibility for a third dose occurs >150 days after receipt of the second dose. Similarly, a threshold of 120 days was used to assess waning VE of a third dose because eligibility for the fourth dose occurs after 120 days. Follow-up time after 120 days from the fourth dose was insufficient to determine VE for this subgroup.

^¶^ VE was estimated by comparing the odds of being vaccinated with 2 and either 3 or 4 doses of a COVID-19 mRNA vaccine in case-patients and control-patients during the BA.1/BA.2 and BA.4/BA.5 periods, calculated as VE = 100 × (1 − odds ratio). Logistic regression models were adjusted for date of hospital admission (biweekly intervals), U.S. Department of Health and Human Services region, age group (18–49, 50–64, and ≥65 years), sex, and race or ethnicity (non-Hispanic White, non-Hispanic Black, Hispanic of any race, non-Hispanic Other, or unknown). Age-specific models were adjusted for age as a continuous variable.

## Discussion

Among immunocompetent adults hospitalized within the IVY Network in 18 states, a monovalent booster dose of mRNA COVID-19 vaccine had limited overall effectiveness against hospitalization caused by currently circulating SARS-CoV-2 Omicron variants, likely because of waning immunity. Waning protection against COVID-19–associated hospitalizations was observed with either 2 or 3 doses of mRNA vaccine during the BA.1/BA.2 period with similar emerging trends during the BA.4/BA.5 periods. These findings demonstrate the importance of staying up to date with COVID-19 vaccinations through receipt of booster doses, which currently consist of bivalent mRNA vaccines for all eligible adults.

Three phenomena likely contributed to the lower overall VE estimated for 3 monovalent mRNA doses during the BA.4/BA.5 period compared with VE during the BA.1/BA.2 period. First, waning protection of mRNA vaccines against COVID-19–associated hospitalizations has been shown previously, and the current findings add to this evidence (*2*,*9*). Although the analysis was stratified by time since last vaccination during each lineage predominance period, the median interval between receipt of the third dose and illness onset during the BA.4/BA.5 period in this analysis was 233 days compared with 145 days during the BA.1/BA.2 period; thus, the BA.4/BA.5 period disproportionately included patients further removed from vaccination, which likely contributed to the lower VE during this period. Waning immunity between lineage periods was less discernible for 2 doses, likely because the median interval between receipt of the second dose and illness onset during the earliest period in this analysis (i.e., BA.1/BA.2) was 277 days, which might already be past the period during which waning can be demonstrated and instead reflects residual protection of 2 doses against COVID-19 hospitalization. In contrast, waning immunity from 4 doses between lineage periods could not be assessed because the median interval from the fourth dose and illness onset during the BA.1/BA.2 and BA.4/BA.5 periods was 26–69 days, which is too recent to show a decrease in protection against COVID-19 hospitalization. Second, increased immune evasion of BA.4/BA.5 lineages has been shown in neutralization assessments and may contribute to lower VE (*10*). However, the extent to which reduced neutralization in vitro correlates with reduced protection against severe disease is unknown; available studies have shown mixed results (*2*–*5*). A study from South Africa showed no difference in VE of 3 monovalent mRNA vaccine doses against hospitalization during the BA.4/BA.5 period compared with the BA.1/BA.2 period at the same intervals from vaccination, which was corroborated by findings from the United Kingdom showing similar VE against BA.2– or BA.4/BA.5–related hospitalizations (*2*,*3*). In contrast, a cohort study in Portugal found reduced protection against severe outcomes during BA.5 predominance (*4*). This was similar to U.S. findings, which indicated that 3-dose VE against hospitalization was lower during the BA.4/BA.5 period compared with the BA.1 period, although these VE estimates did not account for time after the last vaccine dose (*5*). Third, infection-induced immunity in the population substantially increased during and after the BA.1 period. National seroprevalence estimates indicate a 1.8-fold increase in SARS-CoV-2 infections during December 2021–February 2022, with 58% of the U.S. population infected by the end of February 2022.^†††^ Cumulative previous infection during the BA.4/BA.5 period compared with that during the BA.1/BA.2 period likely resulted in a larger proportion of unvaccinated persons having infection-induced immunity during the BA.4/BA.5 period than during the BA.1/BA.2 period; thus, lower VE was observed.

The findings in this report are subject to at least four limitations. First, sample size was insufficient to assess VE varying over time for the BA.2 period separately, resulting in use of a combined BA.1/BA.2 group instead, or to demonstrate substantial waning during the BA.4/BA.5 period. Second, because lineage periods were pooled, the unique contributions of immune evasion associated with each lineage to VE could not be ascertained. Third, because previous infection could not be measured, its effect on VE estimates could only be inferred, not quantified. Finally, follow-up time after the fourth dose to assess waning immunity associated with this dose was insufficient.

Overall, these findings indicate that by the time BA.4/BA.5 lineages became predominant in the United States, effectiveness of 2 or 3 doses of monovalent mRNA vaccines against COVID-19–associated hospitalization had waned. Augmenting population immunity before the winter season through receipt of an updated bivalent COVID-19 booster is important to maximize protection against the predominant BA.5 lineages and prevent COVID-19–associated hospitalizations.

**Summary**

**What is already known about this topic?**

Monovalent mRNA vaccine effectiveness (VE) against COVID-19–associated hospitalization wanes over time; less is known about durability of protection during the SARS-CoV-2 Omicron BA.4/BA.5–predominant period.

**What is added by this report?**

Three-dose monovalent mRNA VE estimates against COVID-19–associated hospitalization decreased with time since vaccination. Three-dose VE during the BA.1/BA.2 and BA.4/BA.5 periods was 79% and 60%, respectively, during the initial 120 days after the third dose and decreased to 41% and 29%, respectively, after 120 days from vaccination.

**What are the implications for public health practice?**

Eligible adults aged ≥18 years should receive an updated bivalent COVID-19 mRNA vaccine to maximize protection against BA.4/BA.5 lineages and to prevent COVID-19–associated hospitalization.

## Footnotes

* These authors contributed equally to this report.

† The IVY Network includes the following hospitals: Barnes-Jewish Hospital (St. Louis, Missouri), Baylor Scott & White Health (Temple, Texas), Baystate Medical Center (Springfield, Massachusetts), Beth Israel Deaconess Medical Center (Boston, Massachusetts), Cleveland Clinic (Cleveland, Ohio), Emory University Medical Center (Atlanta, Georgia), Hennepin County Medical Center (Minneapolis, Minnesota), Intermountain Medical Center (Murray, Utah), Johns Hopkins Hospital (Baltimore, Maryland), Montefiore Medical Center (New York, New York), Oregon Health & Science University Hospital (Portland, Oregon), Ronald Reagan UCLA Medical Center (Los Angeles, California), Stanford University Medical Center (Stanford, California), The Ohio State University Wexner Medical Center (Columbus, Ohio), Vanderbilt University Medical Center (Nashville, Tennessee), UCHealth University of Colorado Hospital (Aurora, Colorado), University of Iowa Hospitals (Iowa City, Iowa), University of Miami Medical Center (Miami, Florida), University of Michigan Hospital (Ann Arbor, Michigan), University of Washington Medical Center (Seattle, Washington), and Wake Forest University Baptist Medical Center (Winston-Salem, North Carolina).

§ On October 12, 2022, the Food and Drug Administration amended the emergency use authorizations of the Moderna COVID-19 vaccine and the Pfizer-BioNTech COVID-19 vaccine to authorize bivalent formulations of the vaccines for use as a single booster dose. The Moderna COVID-19 Vaccine, Bivalent, is authorized for use as a single booster dose in persons aged ≥6 years. The Pfizer-BioNTech COVID-19 Vaccine, Bivalent, is authorized for use as a single booster dose in persons aged ≥5 years. https://www.fda.gov/news-events/press-announcements/coronavirus-covid-19-update-fda-authorizes-moderna-and-pfizer-biontech-bivalent-covid-19-vaccines

¶ COVID-19–like illness was defined as having any one of the following: fever, cough, shortness of breath, loss of taste, loss of smell, new or worsening findings on chest imaging consistent with pneumonia, or use of respiratory support (e.g., high flow nasal cannula, noninvasive ventilation, or invasive mechanical ventilation).

** During the early BA.1 period (December 26, 2021–January 14, 2022), all specimens testing positive for SARS-CoV-2 by RT-PCR were submitted for whole genome sequencing; from January 15, 2022, onward, only specimens testing positive for SARS-CoV-2 by RT-PCR with a cycle threshold <32 for at least one of two nucleocapsid gene targets tested underwent whole genome sequencing. SARS-CoV-2 lineages were assigned by using PANGO on genomes with >80% coverage.

†† Immunocompromising conditions were defined as active solid tumor or hematologic cancer (i.e., newly diagnosed cancer or cancer treatment within the past 6 months); solid organ transplant; bone marrow or stem cell transplant; HIV infection; congenital immunodeficiency syndrome; use of an immunosuppressive medication ≤30 days; splenectomy; or other condition that causes moderate or severe immunosuppression.

§§ Other exclusions included 1) receipt of a non-mRNA vaccine; 2) partial vaccination, including receipt of only 1 mRNA vaccine dose; 3) inability to verify vaccination status; 4) vaccination before CDC recommendations; 5) illness onset >10 days before test date; 6) illness onset >14 days before hospitalization; 7) missing data; and 8) withdrawal from participation.

¶¶ https://www.cdc.gov/vaccines/covid-19/clinical-considerations/archived-covid-19-vacc-schedule.html (Accessed September 27, 2022).

*** 45 C.F.R. part 46.102(l)(2), 21 C.F.R. part 56; 42 U.S.C. 241(d); 5 U.S.C. Sect. 552a; 44 U.S.C. Sect. 3501 et seq.

††† https://covid.cdc.gov/covid-data-tracker/#national-lab (Accessed September 9, 2022).
